# Surgical resection with postoperative stereotactic radiosurgery versus stereotactic radiosurgery alone for large (>3 cm) multiple brain metastases: a propensity score-matched analysis

**DOI:** 10.3389/fonc.2026.1876936

**Published:** 2026-09-03

**Authors:** Xufei Guo, Kuanyu Wang, Shiqi Shi, Li’ao Wang, Yutong Zhao, Dan Xiao, Wang Jia, Shibin Sun, Deling Li

**Affiliations:** 1 Department of Neurosurgery, Beijing Tiantan Hospital, Capital Medical University, Beijing, China; 2 Department of Gamma-Knife Center, Beijing Tiantan Hospital, Capital Medical University, Beijing, China; 3 Beijing Neurosurgical Institute, Capital Medical University, Beijing, China; 4 China National Clinical Research Center for Neurological Diseases (NCRC-ND), Beijing, China

**Keywords:** multiple brain metastases, overall survival, progression-free survival, stereotactic radiosurgery, surgery

## Abstract

**Purpose:**

While surgery plus stereotactic radiosurgery (SRS) is established for solitary brain metastases, its role in multiple brain metastases (MBM) remains unclear. This study compared survival in MBM patients undergoing resection of the largest tumor plus postoperative SRS versus SRS alone.

**Methods:**

This retrospective study included adults with 2–4 MBM, a dominant lesion >3 cm, and Karnofsky Performance Status ≥70. We compared patients undergoing resection of the dominant lesion plus SRS against those receiving SRS alone. Propensity-score matching (PSM) balanced baseline characteristics. Overall survival (OS), progression-free survival (PFS), and prognostic factors were analyzed.

**Results:**

Of 103 patients, 16 received surgery plus SRS and 87 received SRS alone. After PSM, groups were well-balanced (median largest diameter 41.7 vs. 38.4 mm, p=0.218). Surgery plus SRS significantly improved median OS (38.9 vs. 11.6 months, p=0.013) and PFS (14.3 vs. 4.7 months, p=0.048). Multivariable regression identified surgical resection (HR 0.33, 95% CI 0.15–0.71; p=0.004), lung-cancer primary (HR 0.58, 95% CI 0.35–0.97; p=0.037), and targeted/immunotherapy (HR 0.30, 95% CI 0.18–0.51; p<0.001) as favorable prognostic factors, whereas extracranial progression predicted worse survival (HR 2.21, 95% CI 1.32–3.71; p=0.002).

**Conclusion:**

For a highly selected group of MBM patients with good performance status and a dominant mass >3 cm, resecting the largest tumor followed by SRS may be associated with improved OS and PFS compared to SRS alone. Prospective studies are needed to validate these preliminary findings.

## Highlights

- This single-center retrospective cohort study compared resection of the largest brain metastasis plus postoperative stereotactic radiosurgery (SRS) versus SRS alone in patients with 2–4 brain metastases and a dominant lesion >3 cm.
- Propensity score-matched analysis indicated that combined surgery and SRS was associated with improved overall survival (38.9 vs. 11.6 months) and progression-free survival (14.3 vs. 4.7 months).
- Multivariable Cox regression identified surgical resection, lung primary cancer, and receipt of immune/targeted therapy as independent predictors of better survival, while extracranial progression predicted worse outcomes.
- The findings suggest that in selected patients with good performance status and a large dominant metastasis (>3 cm), surgical debulking followed by SRS may offer a potential survival benefit over SRS alone, though these results are preliminary given the limited sample size.
- Prospective studies are needed to validate these results and refine surgical indications for multiple brain metastases.

## 1 Introduction

Brain metastases (BM) develop in 10-40% of patients with advanced solid tumors, most commonly from lung, breast, colorectal cancer, melanoma, and renal cell carcinoma (1, 2). Population-based SEER data (3) indicated that among adult patients diagnosed with brain metastases at the time of primary cancer diagnosis, the median overall survival (OS) remained below 12 months even in the modern era.

The management of brain metastases typically involves surgery, radiation therapy, chemotherapy, immunotherapy, and targeted agents (2, 4, 5). A randomized controlled trial conducted by Patchell et al. (6) demonstrated that surgical resection plus whole-brain radiotherapy (WBRT) prolonged survival of patients with single and accessible brain metastasis. Subsequent adoption of stereotactic radiosurgery (SRS) permitted equivalent local control with less neuro-cognitive toxicity (7), and current guidelines endorse surgery followed by SRS for solitary brain metastases in patients with good performance status and large lesions (maximum diameter > 3–4 cm) (1). In contrast, evidence for surgery in multiple BM (MBM) is restricted to small, single-institution series suggesting benefit only under highly selective conditions (4, 5). Meanwhile, substantial high-level evidence has confirmed the clinical benefit of SRS alone for multiple metastases, particularly in patients with 2–4 lesions (8–12). A prospective multicenter observational study (13) demonstrated that SRS is non-inferior in treating up to 5–10 metastases compared to 2–4 lesions, expanding the indications for SRS. For individual tumors > 3cm within MBM patients, however, single-fraction SRS is limited by radiation necrosis and marginal failure, whereas multi-fraction SRS or surgical debulking may reduce mass effect and permit dose-escalation to residual tumors (14, 15). Direct comparisons between surgical debulking with SRS are lacking, and the surgical indications for MBM remain undefined (16, 17).

We therefore performed a propensity-matched retrospective cohort study to explore the hypothesis that resection of the dominant and significant mass-occupying lesion (> 3 cm) followed by SRS for remaining metastases might be associated with improved survival outcomes relative to SRS alone in a selected cohort of patients with 2–4 BM who are unsuitable for gross-total resection. Here we report patients’ OS, intracranial progression-free survival (PFS), and prognostic factors among 103 patients at single high-volume neurosurgical and radiosurgery centers.

## 2 Materials and methods

### 2.1 Study population

We conducted a single-center, retrospective cohort study of consecutive adult patients (≥ 18 years) who underwent first-course Gamma Knife radiosurgery (GKS) for brain metastases at [Institution blinded for review], between January 2018 and January 2023. Eligibility criteria were (1) Karnofsky Performance Status (KPS) ≥ 70 at the time of GKS, (2) 2 to 4 intracranial metastases with the largest lesion measuring 3–6 cm in maximal diameter and any remaining unresected tumor < 3 cm, and (3) histopathological confirmation of the primary malignancy. Exclusion criteria encompassed prior radiotherapy, recurrent brain metastases, neurological emergency requiring immediate intervention, or an interval > 100 days between craniotomy and SRS for patients with previous resection (18). The final analytic cohort was stratified into two groups: the Surgery + SRS and the SRS-alone groups.

All enrolled patients underwent single-fraction Leksell Gamma Knife radiosurgery. The target delineation and treatment planning were collaboratively performed by a neurosurgeon and a radiation oncologist. For postoperative cavity SRS, the gross tumor volume (GTV) was defined as the contrast-enhancing resection cavity and any residual nodular disease identified on the planning MRI. A standard clinical target volume (CTV) margin of 1–2 mm was typically added to the GTV to account strictly for microscopic infiltration, carefully tailored around critical structures and eloquent cortices. Given the sub-millimeter accuracy of frame-based Gamma Knife radiosurgery, an additional planning target volume (PTV) margin for setup uncertainties was not utilized. For intact (unresected) brain metastases, the GTV was defined strictly as the contrast-enhancing lesion without additional CTV margins.

### 2.2 Data collection

Clinical data for all enrolled patients were extracted from the electronic medical record (EMR) through unique patient identifiers. Demographic variables comprised age at SRS, gender, KPS, primary tumor history, and systemic therapies preceding SRS treatment. Radiological parameters – derived from contrast-enhanced 3 T magnetic resonance imaging (MRI) obtained ≤ 14 days before SRS treatment – included total lesion number, location of the largest metastasis, and maximum tumor diameter. For patients who had undergone tumor resection, surgical variables (craniotomy site and number of metastases removed) were additionally recorded.

Two board-certified neurosurgeons, blinded to treatment allocation, independently conducted standardized telephone follow-up every 3 months after SRS treatment to track patient prognosis and treatment outcomes. The primary follow-up items included: (1) subsequent treatments after discharge, including whole-brain radiation therapy (WBRT), chemotherapy, targeted therapy, and/or immunotherapy; (2) whether intracranial recurrence occurred, along with the site and time of recurrence; (3) patient survival status, and for deceased patients, the cause and date of death; (4) neurological PFS and KPS at the last follow-up for surviving patients. In this study, both OS and PFS were calculated from a common baseline: the date of the initial therapeutic intervention for brain metastases (the date of craniotomy for the surgery plus SRS group, and the date of SRS for the SRS-alone group). In our analysis, PFS specifically refers to intracranial progression-free survival. It was defined as the time from this baseline to the first documented evidence of intracranial progression (including local failure at the treated sites, the emergence of new distant brain metastases, or both) according to the Response Assessment in Neuro-Oncology–Brain Metastases (RANO-BM) criteria (19), or death from any cause, whichever occurred first. Neurological death was adjudicated when central neurological dysfunction was the primary or contributory cause of mortality; all other deaths were classified as non-neurological (20).

### 2.3 Statistical analyses

All statistical analyses were performed with R software (version 4.3.1). 1:3 Propensity score matching (PSM) was conducted using the MatchIt package to mitigate baseline imbalances between the Surgery plus SRS group and the SRS-alone group. Continuous variables were expressed as median and interquartile range (IQR) and compared with the Mann–Whitney U test. Categorical variables were reported as n (%) and compared with Chi-square test or Fisher’s exact test as appropriate.

OS and PFS were estimated by the Kaplan-Meier method and compared between groups with the log-rank test. Independent prognostic factors were identified through multivariable Cox proportional-hazards regression analysis. The proportional hazards assumption was verified using the Schoenfeld residuals test, and no significant violations were observed (p = 0.61). Results were visualized as hazard-ratio (HR) forest plots with 95% confidence intervals (CI). A two-sided p-value of less than 0.05 was considered statistically significant. Reporting follows the STROBE statement for observational studies.

## 3 Results

### ​3.1 Patient enrollment and baseline clinical characteristics

**Figure 1:**
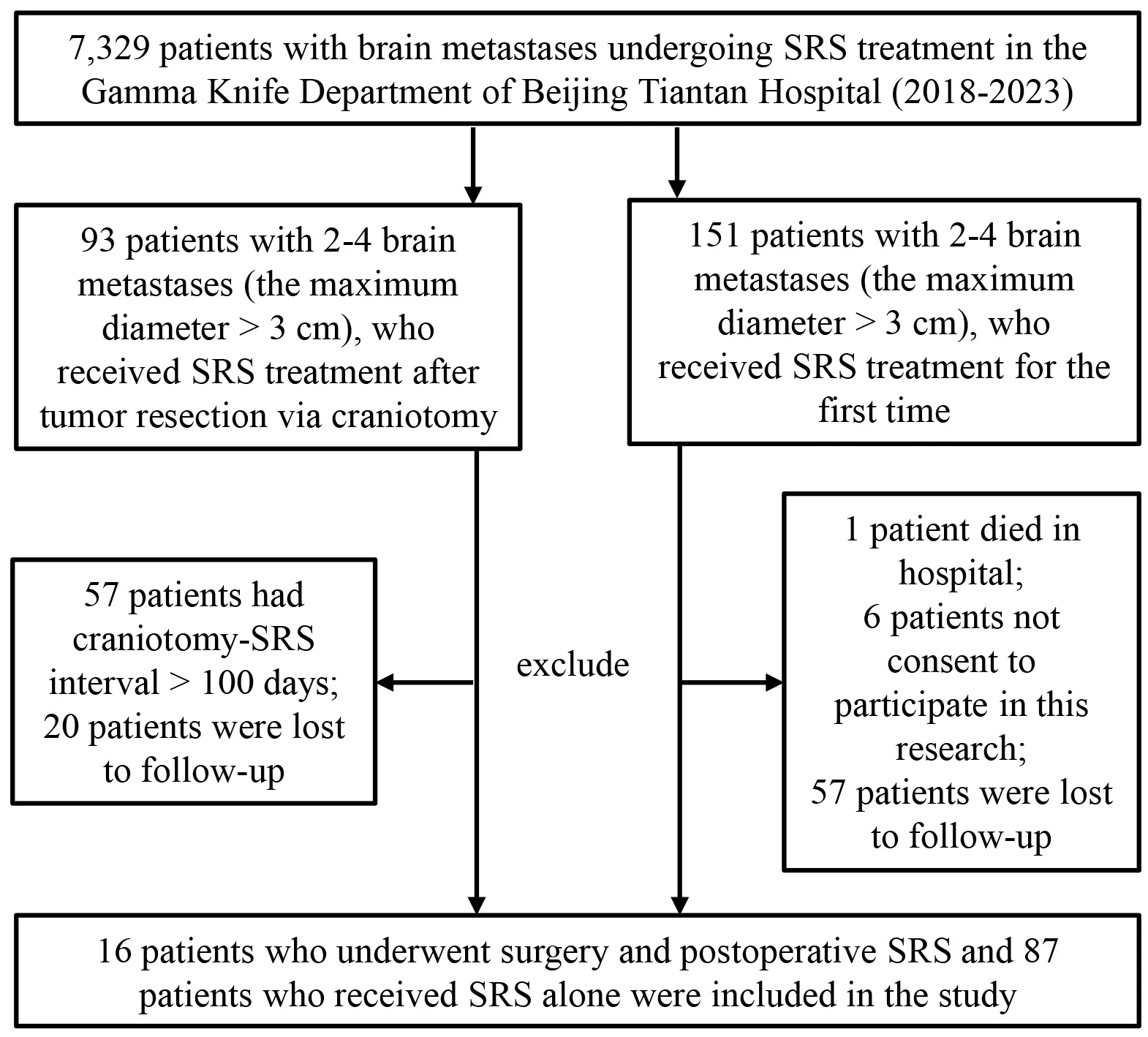
Screening for brain metastases patients cohort.

A total of 103 patients were identified and included in the study, including 16 in the surgery plus SRS group and 87 in the SRS-alone group (**Figure 1**). For patients in the surgery plus SRS group, the median interval between surgical resection and postoperative SRS was 29.5 days (range, 8–76 days). Based on postoperative contrast-enhanced MRI, gross total resection (GTR) of the targeted dominant metastasis was successfully achieved in all 16 patients (100%) in the surgical cohort. Prior to conducting the matching of baseline data, no significant differences were observed between the two groups in terms of age (median: 62.5 vs. 63 years, p = 0.635) or gender (p = 0.208) (**Table 1**). Additionally, there were no significant differences in the number of intracranial tumors, primary tumor type, or tumor location of the largest metastasis. However, the median maximum tumor diameter was significantly larger in the surgery plus SRS group than the SRS-alone group (41.7 mm vs. 36.1 mm, p = 0.008). Regarding post-discharge treatments, no significant differences were found in the rates of chemotherapy or repeated craniotomy between the groups. However, the proportion of patients who received WBRT was higher in the surgery plus SRS group (56.3% vs. 28.7%, p = 0.031), while the SRS-alone group had a higher proportion of patients receiving targeted/immunotherapy (60.9% vs. 18.8%, p = 0.001).

**Table 1:** Baseline characteristics of study patients.

	Original data			Matched data		
Variable	Surgery+SRS# (16)	SRS-alone (87)	P-value	Surgery+SRS (16)	SRS-alone (48)	P-value
Gender			0.208			0.459
Male	5 (31.3)	42 (48.3)		5 (31.3)	20 (41.7)	
Female	11 (68.8)	45 (51.7)		11 (68.8)	28 (58.3)	
Age, y	62.5 (56.3-67.5)	63 (55.5-68)	0.635	62.5 (56.3-67.5)	63 (52.8-67.3)	0.798
KPS*			0.748			0.579
70-80	10 (62.5)	56 (64.4)		10 (62.5)	35 (72.9)	
80-90	6 (37.5)	31 (35.6)		6 (37.5)	13 (27.1)	
RPA**			0.469			0.468
1	8 (50)	35 (40.2)		8 (50)	19 (39.6)	
2	8 (50)	52 (59.8)		8 (50)	29 (60.4)	
No. of brain metastases			0.511			0.920
2	7 (43.7)	46 (52.9)		7 (43.7)	24 (50)	
3	7 (43.7)	25 (28.7)		7 (43.7)	12 (25)	
4	2 (12.5)	16 (18.4)		2 (12.5)	12 (25)	
Primary cancer location						
Lung	11 (68.8)	50 (57.5)	0.398	11 (68.8)	26 (54.2)	0.306
Breast	3 (18.7)	12 (13.8)	0.605	3 (18.7)	9 (18.7)	1.000
Other	2 (12.5)	25 (28.7)	0.174	2 (12.5)	13 (27.1)	0.419
Diameter (largest metastasis, mm)	41.7 (35.8-43.5)	36.1 (32.9-39.4)	**0.008**	41.7 (35.8-43.5)	38.4 (35.1-41.4)	0.218
Location of the largest metastasis			0.264			0.335
Supratentorial	10 (62.5)	66 (75.9)		10 (62.5)	36 (75)	
Infratentorial	6 (37.5)	21 (24.1)		6 (37.5)	12 (25)	

#SRS, Stereotactic radiosurgery; *KPS, Karnofsky performance status; **RPA, Recursive partitioning analysis. Bold values indicate statistical significance (p < 0.05).

To balance baseline characteristics and mitigate the profound confounding effects of subsequent life-prolonging interventions on survival analysis, 1:3 PSM was performed using the following variables: age, gender, number of intracranial tumors, maximum tumor diameter, WBRT, chemotherapy, and targeted/immunotherapy. Although systemic therapies often occur post-surgery, incorporating them into the matching process was a pragmatic clinical decision aimed at isolating the specific survival benefit of surgical resection by ensuring equivalent systemic and whole-brain radiation exposure between the cohorts. The balance of covariates before and after matching was visually evaluated using a Love plot based on absolute standardized mean differences (SMD) (**Supplementary Figure 1**). After matching, 16 patients remained in the surgery plus SRS group and 48 in the SRS-alone group. All baseline characteristics were well-balanced with no significant differences after matching (**Table 1**). The two groups were comparable in terms of maximum tumor diameter (41.7 mm vs. 38.4 mm, p = 0.218), WBRT (56.3% vs. 43.8%, p = 0.385), and targeted/immunotherapy (18.8% vs. 31.3%, p = 0.335).

Regarding the dosimetric parameters of the dominant lesion, the median marginal prescription dose was significantly higher in the surgery plus SRS group compared to the SRS-alone group (15.0 Gy [range, 12–16 Gy] vs. 12.0 Gy [range, 7–16 Gy], p < 0.001). The corresponding median central maximum doses were 29.5 Gy and 24.0 Gy, respectively (p < 0.001). Both cohorts were prescribed to a median isodose line of 50% (p = 0.111). For the remaining smaller intracranial metastases, which were concurrently treated during the same radiosurgical session, marginal doses were individually tailored, typically ranging from 15 to 24 Gy depending on tumor volume and proximity to critical structures.

### 3.2 Outcomes

As shown in **Table 2**, the median follow-up times were 47.2 months (IQR 32.4–51.6) and 46.6 months (IQR 29.7–60.5) for the two groups, respectively (p = 0.515). Before matching, there was no significant difference in overall mortality between the groups (56.3% vs. 74.7%, p = 0.131). The overall intracranial recurrence rate (including local, distant, or both) also did not differ significantly (81.3% vs. 59.8%, p = 0.101). The 1-year recurrence rate was comparable between the two groups (43.8% vs. 46.0%, p = 0.869). However, the 1-year mortality was significantly lower in the surgery plus SRS group (12.5% vs. 41.4%, p = 0.027). No significant differences were observed in the death reasons (neurological death: 55.6% vs. 44.6%, p = 0.537), or the median KPS among surviving patients (70 vs. 90, p = 0.128).

**Table 2:** Follow-up treatment and prognosis of patients.

	Original data			Matched data		
Variable	Surgery+SRS# (16)	SRS-alone (87)	P-value	Surgery+SRS (16)	SRS-alone (48)	P-value
Follow up, months	47.2 (32.4-51.6)	46.6 (29.7-60.5)	0.515	47.2 (32.4-51.6)	46.8 (31.8-59.9)	0.381
Subsequent treatment						
WBRT*	9 (56.3)	25 (28.7)	**0.031**	9 (56.3)	21 (43.8)	0.385
Chemotherapy	11 (68.8)	54 (62.1)	0.610	11 (68.8)	31 (64.6)	0.761
Immune/Targeted therapy	3 (18.8)	53 (60.9)	**0.001**	3 (18.8)	15 (31.3)	0.335
Repeat surgery	2 (12.5)	4 (4.6)	0.214	2 (12.5)	3 (6.3)	0.419
Recurrence rate (1 year)			0.869			0.470
Local	3 (18.8)	15 (17.2)		3 (18.8)	10 (20.8)	
Distant	1 (6.3)	9 (10.3)		1 (6.3)	6 (12.5)	
Local and distant	3 (18.8)	16 (18.4)		3 (18.8)	10 (20.8)	
Overall recurrence	13 (81.3)	52 (59.8)	0.101	13 (81.3)	31 (64.6)	0.212
Mortality (1 year)	2 (12.5)	36 (41.4)	**0.027**	2 (12.5)	24 (50)	**0.008**
Overall mortality	9 (56.3)	65 (74.7)	0.131	9 (56.3)	41 (85.4)	**0.014**
Death reason			0.537			0.616
neurological death	5 (55.6)	29 (44.6)		5 (55.6)	19 (46.3)	
non-neurological death	4 (44.4)	36 (55.4)		4 (44.4)	22 (53.7)	
Follow-up KPS**	70 (70-80)	90 (80-100)	0.128	70 (70-80)	90 (65-95)	0.259

#SRS, Stereotactic radiosurgery; *WBRT, Whole brain radiotherapy; **KPS, Karnofsky performance status. Bold values indicate statistical significance (p < 0.05).

**Figure 2:**
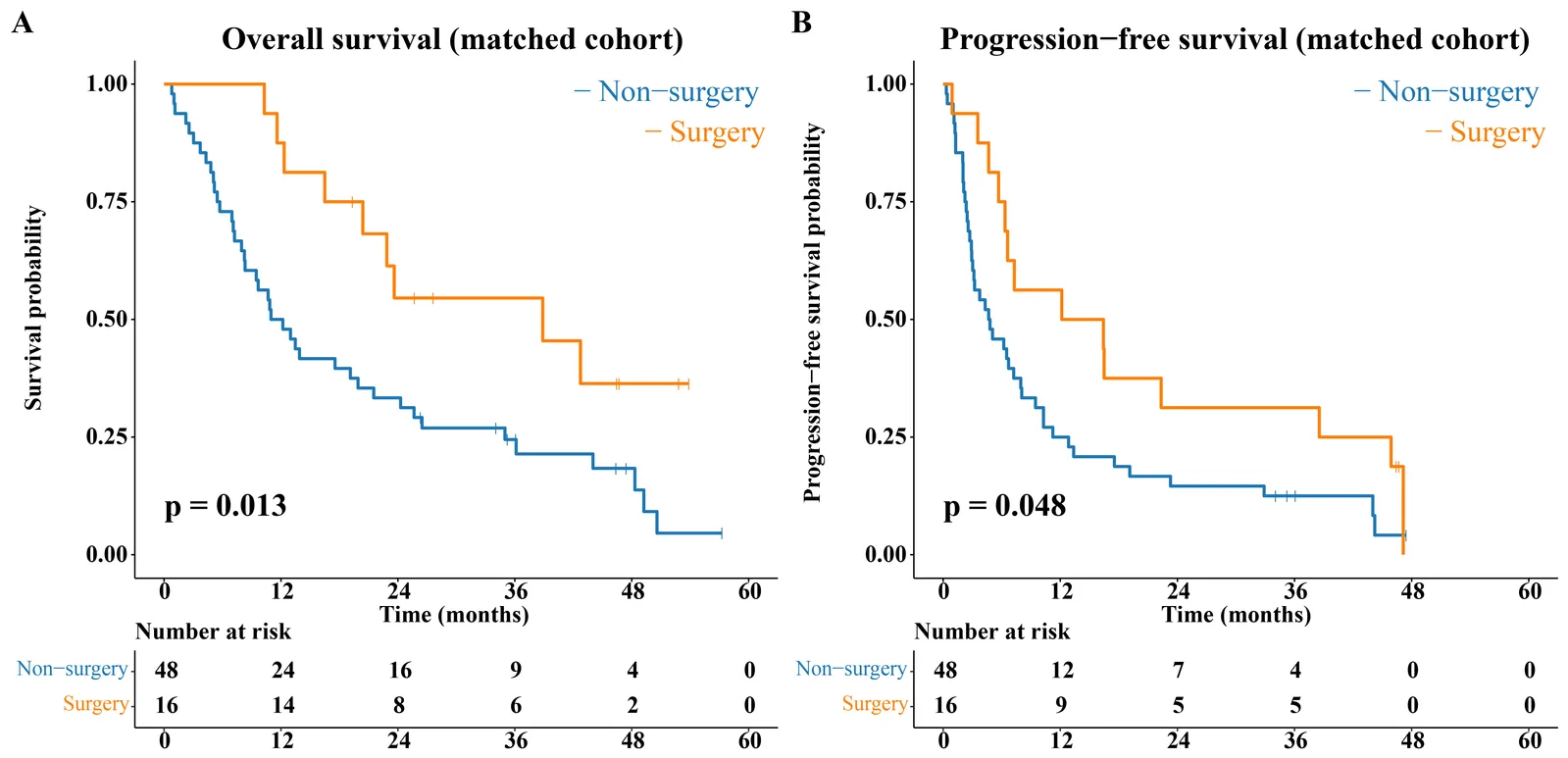
Survival and progression-free survival curves of matched patients. **(A)** Overall survival curve for both groups of patients; **(B)** Progression-free survival curve.

After PSM, the overall mortality was significantly higher (85.4% vs. 56.3%, p = 0.014) in the SRS-alone group compared to the surgery plus SRS group, as well as 1-year mortality (50.0% vs. 12.5%, p = 0.008). Kaplan-Meier analysis revealed that after PSM matching, the median OS was significantly longer in the surgery plus SRS group than in the SRS-alone group (38.9 months [95% CI: 20.4–NR] vs. 11.6 months [95% CI: 8.3–21.5], p = 0.013). The corresponding OS rates at 6, 12, and 24 months were 100%, 87.5%, and 54.5% in the surgery plus SRS group, compared to 72.9%, 50.0%, and 33.3% in the SRS-alone group. Furthermore, the median PFS was also significantly prolonged in the surgery plus SRS group (14.3 months [95% CI: 6.4–NR] vs. 4.7 months [95% CI: 3.0–8.1], p = 0.048). The 6-, 12-, and 24-month PFS rates for the combined treatment cohort were 75.0%, 56.2%, and 31.2%, respectively, versus 45.8%, 25.0%, and 14.6% for the SRS-alone cohort (**Figure 2**). Furthermore, a sensitivity analysis restricting the PSM criteria strictly to pretreatment baseline variables confirmed that the robust survival advantage of the surgery plus SRS cohort remained statistically significant (OS: p = 0.048; PFS: p = 0.029).

### 3.3 Independent risk factors of poor prognosis

**Figure 3:**
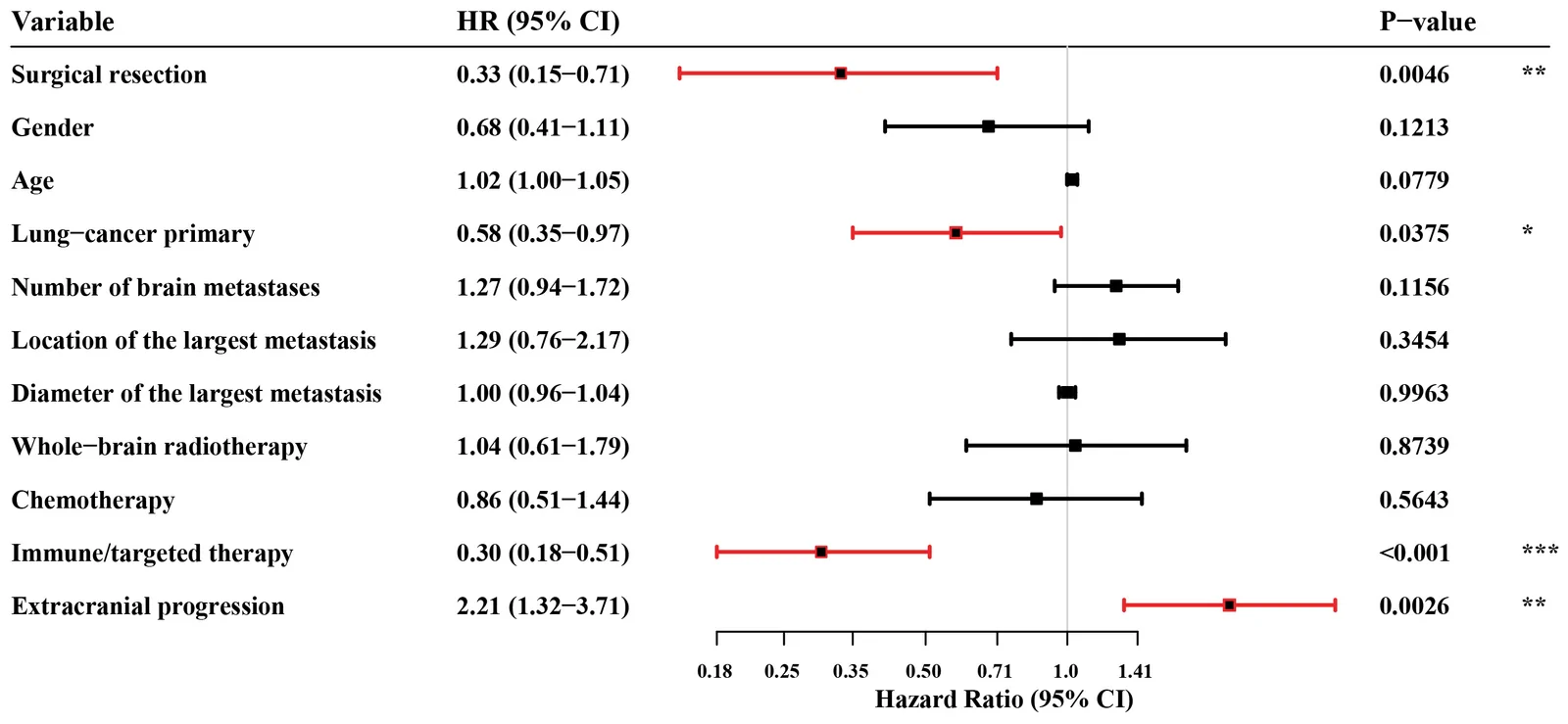
Multivariate COX regression analysis of factors influencing survival in all patients.

Multivariable Cox regression analysis was performed including all patients and identified four variables independently associated with overall mortality (**Figure 3**). After adjustment for surgical resection, gender, age, lung-cancer primary, number of brain metastases, location of the largest metastasis, diameter of the largest metastasis, whole-brain radiotherapy, chemotherapy, immune/targeted therapy, and extracranial progression, surgical resection of the largest brain metastasis (HR 0.33, 95% CI 0.15–0.71; p = 0.004), a lung-cancer primary (HR 0.58, 95% CI 0.35–0.97; p = 0.037), and receipt of targeted and/or immunotherapy (HR 0.30, 95% CI 0.18–0.51; p < 0.001) were independently associated with improved survival. By contrast, concurrent extracranial progression during follow-up remained an independent predictor of death (HR 2.21, 95% CI: 1.32–3.71; p = 0.002).

Given the relatively large number of covariates in the primary model, a simpler sensitivity Cox model was constructed, restricted to the four most significant prognostic drivers (surgical resection, lung-cancer primary, targeted/immunotherapy, and extracranial progression) to prevent overfitting. In this parsimonious model, surgical resection remained a robust independent predictor of improved overall survival (HR 0.36, 95% CI: 0.17–0.75; p = 0.006).

## 4 Discussion

Optimal management for patients with MBM who harbor at least one large lesion (> 3 cm in diameter) with significant mass effect remains unresolved. Surgical resection is unequivocally beneficial for single brain metastases (6, 17, 21), but its role in the context of MBM is controversial and poorly defined (5, 17, 22–26), with existing evidence dominated by smaller tumors (16, 27). SRS is the current standard for most patients with MBM; however, RTOG 90–05 study showed that local control declines and radionecrosis rises steeply when lesions exceed 3 cm (28). Whether surgery of the dominant mass plus SRS to remaining foci improve survival over SRS alone in MBM patients is therefore unknown. To fill this evidence gap, we conducted a comparative cohort study restricted to MBM patients with one metastasis > 3 cm. Our exploratory analysis suggests that resection of the largest metastasis followed by adjuvant SRS was associated with prolonged OS and PFS compared to SRS alone in our specific study population.

This study restricted enrollment to patients with MBM harboring one large (> 3 cm in diameter) space-occupying lesion. After craniotomy-directed cytoreduction, participants received Gamma-Knife SRS (surgery plus SRS group); controls received SRS alone. Baseline characteristics were well balanced following PSM. Median maximum tumor diameter was 41.7 mm versus 38.4 mm in the surgery plus SRS and SRS-alone groups, respectively (p = 0.218). At 1 year, mortality was lower (12.5% vs. 50.0%). By the study close, the cumulative death rate remained lower in the surgery plus SRS group (56.3% vs. 85.4%). Although there was no significant difference in recurrence rates between the two groups, PFS was significantly longer in patients undergoing surgery plus SRS group (p = 0.048).

A critical consideration in MBM survival analysis is the heterogeneity of primary tumor biology, particularly regarding driver mutations (e.g., EGFR/ALK in lung cancer). While explicit genomic data were not available for all patients in this retrospective series, we incorporated “receipt of targeted/immunotherapy” as a matching variable in our PSM analysis. In modern oncological practice, the administration of targeted agents serves as a clinical proxy for the presence of actionable driver mutations. By balancing this variable (18.8% vs. 31.3%, p=0.335 after matching), we aimed to mitigate the confounding effect of favorable tumor biology on survival outcomes, suggesting that the observed benefit of surgery is not solely attributable to systemic therapy responsiveness. Similarly, although a relatively high proportion of patients eventually required WBRT as a salvage treatment for subsequent distant progression, matching for WBRT exposure effectively mitigated its potential confounding effects on both survival and intracranial control comparisons.

Surgical intervention for MBM remains inadequately studied. Uma et al. (23) highlighted in a systematic review that the average publication year for studies on surgical resection of MBM was 2004, indicating a scarcity of recent evidence. Outcomes after surgery alone for MBM patients have been reported in several studies. In a landmark study by Bindal et al. (29) (1993), 56 MBM patients were included; 30 patients had one or more lesions left unresected (Group A) and 26 patients had all lesions removed (Group B), compared with 26 matched patients with single brain metastasis who underwent resection (Group C). Results showed no significant difference in OS between Groups B and C (p > 0.5), with both groups outperforming Group A. Similarly, Maurizio et al. (30) compared 32 MBM patients undergoing surgical resection of all lesions with 30 matched patients with single brain metastasis. They found no significant difference in median OS (14.6 vs. 17.4 months, p = 0.2). A retrospective study by Stephanie et al. (31) of 216 brain metastases patients, including 87 with multiple metastases, reported that the number of intracranial lesions did not affect OS on univariate analysis (p = 0.844). Together, these studies suggest that resection of all lesions can lead to favorable outcomes in a select subgroup of patients with limited number of metastases and good performance status. However, they do not address whether surgical intervention provides any benefit for patients with MBM that are not amenable to complete resection.

Several studies have investigated the combination of surgical resection and SRS for patients with MBM. Maria et al. (16) included 129 MBM patients with dominant lesions (>2 cm), comparing 84 patients who underwent surgery plus SRS and 45 patients treated with SRS alone. No significant difference in mortality was observed between the groups during follow-up (69.1% vs. 77.8%, p = 0.292), leading the authors to recommend SRS for all intracranial lesions in patients with dominant metastatic lesions. However, notable baseline imbalances were noted between the groups, including differences in maximum tumor diameter (31 mm vs. 21 mm, p < 0.001) and the use of adjuvant radiotherapy (78.6% vs. 40.0%, p < 0.001), which substantially compromise the reliability of the conclusions. Furthermore, the smaller tumor diameter in the SRS-alone group suggests a potential selection bias toward treating lesions more amenable to SRS. In contrast, Varun et al. (27) reported the largest cohort to date on postoperative SRS for MBM. Compared with SRS alone, surgical resection was associated with reduced mortality (HR 0.67, p = 0.005). Nonetheless, the majority of tumors in the SRS-alone group were relatively small, with only 7 (1.3%) exceeding 3 cm in diameter. Although PSM was performed and yielded similar conclusions, the mean tumor diameter in both groups after matching was 2.4 cm—still below 3 cm—raising questions regarding the generalizability of these findings to patients with larger lesions. Therefore, no well-designed studies have compared the efficacy of surgical resection plus SRS versus SRS alone in patients with MBM harboring at least one lesion >3 cm in diameter.

The incidence of brain metastases is continuously rising. This rise can be attributed to two primary factors: advances in systemic therapies, which have improved control of primary extracranial disease and prolonged overall patient survival (22, 32–37), and refinements in MRI technology, which have enhanced the detection of small intracranial tumors (17, 23, 38). It is noteworthy that patients with MBM outnumber those with solitary lesions (22, 39–41). Within this population, the challenge of managing large, dominant lesions is increasingly encountered in clinical practice. Reduction of intracranial tumor burden has been proven to significantly improve neurocognitive function, quality of life, and overall survival; therefore, the primary therapeutic goal for patients with brain metastases is to achieve meaningful tumor volume reduction. Importantly, this surgical debulking not only relieves local mass effect but also provides a crucial dosimetric advantage. In our cohort, we were able to deliver a significantly higher median marginal dose to the postoperative cavity compared to the intact large tumors (15.0 Gy vs. 12.0 Gy). While intact tumors >3 cm necessitate dose de-escalation to mitigate the risks of radiation necrosis and severe edema, prior surgical resection allows for the safer administration of more tumoricidal radiation doses. Therefore, the prolonged overall and progression-free survival observed in the surgery plus SRS group may be closely related to this capacity for postoperative dose escalation. Our findings, which suggest a survival benefit for surgical resection in this specific setting, therefore hold relevance for refining treatment strategies in this evolving landscape.

### 4.1 Limitations

This study has several limitations. First and foremost, the sample size of the surgical cohort (n=16) is small, which limits statistical power and precludes extensive subgroup analyses. This limited number reflects the strict inclusion criteria (dominant lesion >3 cm, oligometastatic disease, good KPS), representing a rare but clinically challenging subset of patients. Consequently, our findings should be interpreted as hypothesis-generating rather than definitive. Second, potential selection bias regarding tumor location cannot be fully excluded. Although we matched for supratentorial vs. infratentorial location, surgical candidates may have harbored more accessible lesions (e.g., non-eloquent cortex), whereas patients in the SRS-alone group might have had lesions in deep or eloquent areas that discouraged resection, despite having similar KPS. This unmeasured confounder is inherent to retrospective surgical studies. Third, as a single-institution study, the findings may not be generalizable to other centers due to potential differences in patient demographics, surgery quality, etc. Fourth, although our study selected a more appropriate SRS control group, its retrospective design inherently introduces potential confounding factors. Specifically, we were unable to consistently capture and match for several highly relevant clinical and biological parameters—such as the detailed symptomatic presentation, exact steroid requirements, baseline extracranial disease burden, patient surgical fitness, precise molecular status (e.g., actionable driver mutations), and specific radiographic features like the degree of mass effect, midline shift or cystic versus solid morphology—due to incomplete historical medical records. Although we matched patients based on maximum tumor diameter, KPS, and the receipt of targeted/immunotherapy to partially account for tumor burden, clinical status, and systemic disease control, unmeasured confounding related to these specific variables may still influence treatment selection and survival outcomes. Fifth, the lack of volumetric tumor analysis, detailed radiosurgical dosimetric parameters, and standardized neurocognitive function assessment represents a limitation. Although volumetric analysis may offer greater precision for assessing tumor burden, the use of maximum linear diameter in our study aligns with established clinical and research standards. Indeed, the Response Assessment in Neuro-Oncology Brain Metastases (RANO-BM) criteria (19), as well as pivotal phase 3 randomized controlled trials guiding postoperative SRS (such as those by Brown et al. (7) and Mahajan et al. (42)), utilize maximum diameter for tumor or cavity evaluation. Additionally, due to historical data archival constraints and software upgrades, granular 3D dosimetric data—such as the gradient index, V12Gy to normal brain tissue, and specific dose constraints for organs at risk—could not be systematically retrieved to further evaluate potential radiation-related toxicity. Furthermore, comprehensive longitudinal neurocognitive testing was not routinely performed in this historical cohort, limiting our evaluation of these specific endpoints. Sixth, the inability to systematically evaluate and compare treatment-related morbidity constitutes another significant limitation. Granular safety outcomes—such as specific surgical complications, the precise incidence of symptomatic radiation necrosis, nuances of neurological deterioration, and long-term steroid dependence—were inconsistently documented in historical electronic medical records.

Consequently, our study primarily focused on survival metrics. Future validation through multi-center, prospective cohorts or randomized controlled trials is urgently needed to rigorously assess both the comparative safety and quality-of-life impacts, as well as the definitive survival benefits of these two treatment strategies.

## 5 Conclusion

This retrospective cohort study suggests that in a highly selected group of patients with good performance and MBM featuring a significant mass (>3 cm), resection of the largest tumor followed by SRS may be associated with improved overall survival and progression-free survival compared with SRS alone. These preliminary findings support the rationale for future multi-center prospective studies to provide higher-level evidence and refine surgical indications for MBM.

## Data Availability

The raw data supporting the conclusions of this article will be made available by the authors, without undue reservation.

## Supplementary material

Supplementary Figure 1 Love plot displaying the absolute standardized mean differences (SMD) of covariates before and after PSM. A vertical dashed line is placed at SMD = 0.1.
